# Numerical simulation study on the stability of coal pillar supported by end gang mining under the effect of blasting vibration

**DOI:** 10.1038/s41598-025-02733-1

**Published:** 2025-05-25

**Authors:** Juyu Jiang, Yulong Zhang, Dong Wang, Lanzhu Cao, Laigui Wang, Changbo Du

**Affiliations:** 1 College of Mining, Liaoning Technical University, Fuxin, 123000 Liaoning China; 2 School of Mechanics & Engineering, Liaoning Technical University, Fuxin, 123000 Liaoning China; 3 School of Civil Engineering, Liaoning Technical University, Fuxin, 123000 Liaoning China

**Keywords:** Blasting vibration, Highwall mining, Coal pillar, Parameter design, Stability analysis, Coal, Fossil fuels, Engineering

## Abstract

The stability of the supporting coal pillar is crucial to determining the safe and efficient popularization of highwall mining technology. In the meanwhile, the repeated and prolonged impact of mine blasting on the supporting coal pillar cannot be overlooked in highwall mining. Therefore, it is essential to propose a technically feasible parameter design scheme for supporting coal pillars under blasting vibration, taking into account specific circumstances. The attenuation law of blasting seismic waves is analyzed through in-situ vibration measurement in the south side of an open pit mine. A numerical simulation model is established to compare and analyze the vibration data obtained from numerical simulation with vibration measurement data, which verified the accuracy of the numerical simulation and determined the response law of blasting vibration. Based on instability and failure criteria for supporting coal pillars, a design scheme for supporting coal pillar parameters under different single charge amounts and blasting distances is proposed. The research findings show that peak velocity of blasting seismic wave is higher in horizontal radial and vertical blasts than in horizontal tangential blasts; there exists a nonlinear negative correlation between peak blast vibration velocity and distance from blast center; vertical attenuation velocity is faster than horizontal direction; main vibration frequency concentrates between 5-15 Hz, decreasing continuously with the increase of detonation center distance. Furthermore, based on the instability and failure criteria of supporting coal pillars and the limit caving distance of supporting coal pillar, the design scheme of supporting coal pillar width is optimized. The reasonable width of the supporting coal pillar is positively and nonlinearly correlated with single charge amount, negatively and nonlinearly correlated with blasting center distance.

## Introduction

It is estimated that the total amount of coal resources covered by end slope is more than 10 billion tons in domestic open-pit coal mines, due to the limitation of slope stability, stripping ratio, transportation system layout and mining boundary. The amount of coal covered by end slope can reach 20% of the total amount of recoverable geological coal in the survey area in some open-pit mines, including many high-quality coal types, resulting in serious waste of resources^1,2^. Compared with other mining methods such as coordinated mining and room-pillar blasting mining in adjacent open-pit mines, recover coal resources by end-wall shearer have the advantages of low cost, quick effect and high recovery rate, and has been gradually promoted and applied. When the end-slope mining technology is used to mine the end-slope coal, the stability of the supporting coal pillar plays a decisive role in the overall stability of the end-slope, while the stability of the supporting coal pillar depends on the reasonable design of the width of the supporting coal pillar. Therefore, experts and scholars at home and abroad have conducted a lot of research on the stability of supporting coal pillars for end-slope mining. Shi Zhiyuan^3^ used the method of combining theory with FLAC 3D numerical simulation to analyze the instability failure mechanism and dynamic change law of supporting coal pillar under the influence of mining depth and the width of supporting coal pillar; Porathur^4^ et al. used FLAC 3D to study the variation of slope stability with the width of supporting coal pillar and mining width in open-pit coal mine; Chandar^5^ et al. analyzed the influence of mining depth, width of supporting coal pillar and number of end-slope mining chambers on slope stability through numerical simulation; JIANG J^6^ et al. established a three-dimensional harmonic vibration response model for web pillars, investigating the influence of blasting parameters including single-shot explosive charge, elevation difference, and horizontal blast distance on the maximum instantaneous dynamic stress of pillars. Their study revealed the mechanism of blasting dynamic load effects on plastic zone width and stability of web pillars; DING X^7^ et al. simulated the entire process of highwall mining using discrete element simulation software. Through monitoring and analyzing the stress and deformation characteristics of web pillars, they constructed vertical load distribution models for pillars under three distinct roadway depths and established a mechanical analysis model for surrounding rock in highwall mining roadways; TAN Y^8^ et al. proposed a sustainable multi-layer stratified mining method for highwall operations. By simplifying the interlayer between upper and lower roadways as a beam structure model, they analyzed the bending moment distribution characteristics under highwall miner loading and developed a computational methodology for determining optimal interlayer thickness ranges.; Roy S^9^ et al. developed a 3D discrete element model incorporating jointed overlying rock masses for highwall panel analysis using 3DEC software. Their research yielded recommendations for optimized and safety-driven design of highwall mining panels.; Wu H^10^ et al. formulated a bearing capacity model for pillars in highwall mining that comprehensively accounts for dynamic loads from mining vehicles and static loads from overlying strata. This culminated in the derivation of a criterion equation for pillar stability evaluation.

At present, experts and scholars at home and abroad mainly focus on the stability of supporting coal pillars in end-wall mining under static load conditions. However, due to the characteristics of open-pit mining, the impact loads such as blasting and stripping of the working side and periodic vibration of the transportation system will cause lasting and repeated disturbances to the supporting coal pillars, which will reduce the strength parameters of the supporting coal pillars in the end-wall mining, and increase the damage range of the plastic zone, leading to affects the stability of the supporting coal pillars in the end-wall mining. Therefore, the influence of blasting vibration on the stability of supporting coal pillars can not be ignored. In order to mining the coal resources covered by the end-slope of the open-pit mine safely and efficiently in China, this paper takes an open-pit mine end-slope mining project as the background. Through numerical simulation, analyzed the influence of blasting dynamic load on the stability of supporting coal pillars in end-slope mining, and the parameter design scheme of supporting coal pillars in end-slope mining of the open-pit mine under different blasting conditions is proposed.

## Analysis of blasting seismic wave propagation and attenuation laws

### Study on the background

The research area of this paper is the south side of an open-pit mine. The main coal seams are 4# and 9# coal seams. The coal-bearing strata are nearly horizontal, the rock mass integrity is good, and the engineering geological and hydrogeological conditions are simple. The end-wall mining of 4# coal seam is designed. The physical and mechanical parameters of coal and rock strata in this study area is shown in Table 1. After the construction of the mining chamber is completed, the inner dump is followed up in time, and the slope stability is improved by the inner dump pressure foot.

**Table 1:** Physical and mechanical parameters of coal and rock mass.

Lithology	Average thickness (m)	Volumetric weight (kN·m^−3^)	Elastic modulus (MPa)	Poisson ratio	Cohesion (MPa)	internal friction angle (°)	compressive strength (MPa)	tensile strength (MPa)
Loess	36	19.5	8.6	0.31	0.028	22	1.8	0.0125
Sand mudstone	133	24.6	2850	0.28	0.87	33	65	3.2
4#Coal	11.9	14.1	1500	0.31	0.36	36.8	8.8	0.79
Sandstone	100	25.2	5425	0.2	1.1	35	80	4.62

Because the research area is the end slope that has been formed in open-pit mining, the internal drainage tracking will be carried out immediately after the end of mining, so the natural factors will not be the main influencing factors of the stability of the supporting coal pillar in the end slope mining. Considering the influence of engineering geological conditions, resource recovery rate, mining boundary conditions and technical equipment, the height of the supporting coal pillar is determined to be 5 m, the mining width is 3.3 m, and the mining depth is 150 m as the quantitative parameters of the end slope mining scheme. The width of the supporting coal pillar is the main design engineering variable. Due to the long-term use of blasting for stripping and mining in the field, the influence of external disturbance factors on the stability of supporting coal pillars cannot be ignored. By analyzing the propagation and attenuation law of blasting seismic waves, the influence degree of blasting seismic waves on the stability of supporting coal pillars in end-wall mining can be determined, which provides a basis for the reasonable supporting coal pillars width design.

### Design and analysis of blasting seismic wave monitoring scheme

**Fig.1:**
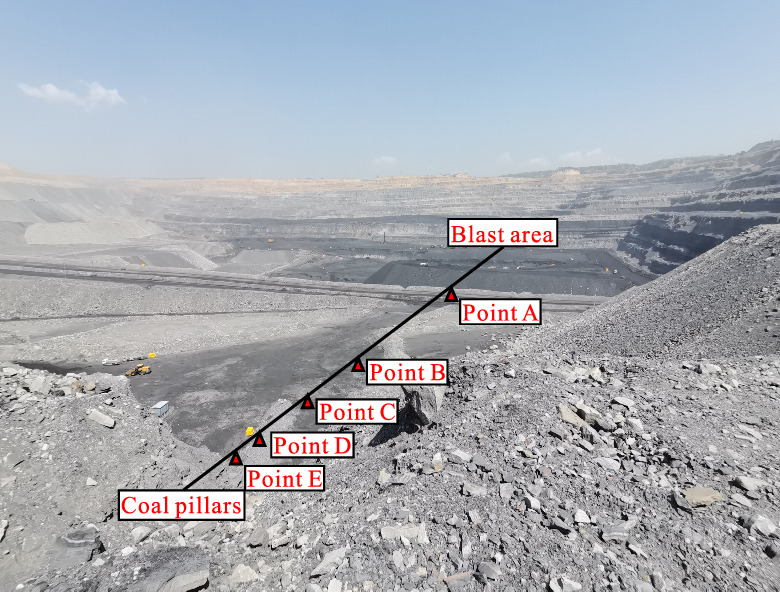
Actual burial of site monitoring points.

**Fig. 2:**
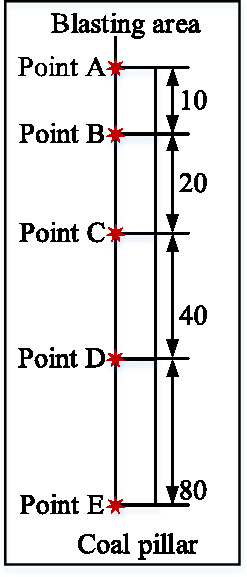
Design of monitoring point spacing.

In order to analyze the propagation and attenuation law of blasting seismic wave and clarify the effect of blasting load on the stability of supporting coal pillar in end mining. The supporting coal pillar is set up in the eastern end area of the south slope. According to the spacing of 10 m, 20 m, 40 m and 80 m, five monitoring points A, B, C, D and E are arranged to monitor at the same time. The schematic diagram of the layout of the monitoring points and the design diagram of the monitoring distance are shown in Fig. 1 and Fig. 2.

Based on the five monitoring points laid out in Fig. 2, the blasting data under different blasting parameters were collected, and the collected data are shown in Table 2. The blasting method adopts multi-row hole-by-hole millisecond blasting. As shown in Table 2, the collected information of 5 blasting operations is counted, a total of 25 sets of effective data.

**Table 2:** Statistics of vibration measurement data.

Times	Single blasting volume /kg	Point-weighted	blasting center distance /m	Vibration peak velocity /cm·s^−1^			resultant velocity /cm·s^−1^	vibration frequency /Hz		
				*V*_x_	*V*_y_	*V*_z_		*F*_x_	*F*_y_	*F*_z_
No.1	420	A	750.65	0.3051	0.216	0.2621	0.4778	8.7	10.5	12.9
		B	760.65	0.3355	0.1891	0.2238	0.453	8.8	8.6	8
		C	780.65	0.1926	0.1079	0.1758	0.3418	8.9	20.8	8.6
		D	820.65	0.1075	0.0983	0.126	0.1984	9.1	10.5	8.6
		E	900.65	0.1044	0.0657	0.1074	0.1636	7.6	8.2	8.6
No.2	350	A	1759.46	0.0391	0.0248	0.0285	0.0483	3.6	3.3	4.1
		B	1769.46	0.0406	0.0264	0.0269	0.0505	3.3	4	3.8
		C	1789.46	0.0399	0.0210	0.0258	0.0463	3.6	3.6	5.4
		D	1829.46	0.0375	0.0207	0.0235	0.0408	8.2	5.5	6.1
		E	1909.46	0.035	0.0224	0.0167	0.0365	6.4	6.4	6.8
No.3	380	A	2010.52	0.0925	0.074	0.0919	0.1499	9.6	8.8	14
		B	2020.52	0.0852	0.0942	0.082	0.1448	11.2	13.1	12
		C	2048.52	0.0688	0.0736	0.0522	0.1135	10.9	9	14
		D	2080.52	0.0576	0.0532	0.0513	0.0879	9.6	11	10.4
		E	2160.52	0.047	0.0423	0.0298	0.0619	8.9	7.8	11
No.4	480	A	2010.11	0.0765	0.0382	0.0753	0.1139	7.7	6.7	5.2
		B	2030.11	0.0887	0.0396	0.0682	0.1187	8	9.7	6.2
		C	2050.11	0.0685	0.0390	0.0629	0.1008	6.7	5	5
		D	2190.11	0.0566	0.0336	0.0594	0.0887	8	6.1	5
		E	2270.11	0.0413	0.0289	0.0407	0.0648	6.6	6	6.4
No.5	300	A	2461.07	0.0489	0.0334	0.0324	0.0607	4.5	9.4	4.8
		B	2471.07	0.0544	0.0469	0.0322	0.0787	9	6.4	9.9
		C	2491.07	0.0426	0.0269	0.0288	0.0511	6.8	4.4	4.3
		D	2531.07	0.035	0.0264	0.0286	0.0504	5	4.9	5.4
		E	2611.07	0.0306	0.0169	0.0252	0.0417	7.7	6.5	12.2

The data in Table 2 show that the peak vibration velocity and the main vibration frequency generally show an attenuation trend, with the increase of the explosion center distance. The main vibration frequency of blasting seismic wave is mainly concentrated between 5 Hz ~ 15 Hz. According to the existing research results of relevant scholars, the natural frequency of rock slope is usually low, generally between 2 Hz ~ 10 Hz^11^. The main vibration frequency of the blasting seismic wave monitored is mostly within the natural vibration frequency range of the slope, which may cause resonance phenomenon and affect the structural integrity of the rock mass, reduce the strength of the supporting coal pillar, and increase the instability risk of the supporting coal pillar. Therefore, when evaluating the influence of blasting seismic wave on the support coal pillar of end slope mining, attention should be paid to the change of blasting seismic wave frequency.

According to the Sadovsky formula^12^, the formula of vibration velocity peak attenuation law of blasting seismic wave can be expressed as:

1 $$V = K\left( {\frac{{\sqrt[3]{Q}}}{R}} \right)^{\alpha }$$

where *Q* is the amount of detonating charge or a single section of the maximum amount of detonating charge (kg), *V* is the vibration velocity (cm/s), *R* is the distance between the burst centre (m), *K* and *α* are coefficients reflecting the degree of seismic wave attenuation^13^.

**Fig. 3:**
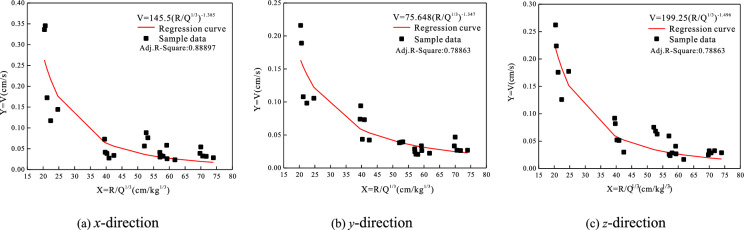
Attenuation pattern and regression curve of horizontal radial velocity.

Based on the Sadovsky formula, the peak velocity of vibration was fitted in the horizontal radial *x*, horizontal tangential *y* and vertical *z* directions of the monitoring points in Table 2 by Origin software, then the propagation attenuation law and regression curve of the blasting seismic wave were obtained. As shown in Fig. 3, the peak value of blasting vibration velocity is exponentially negatively correlated with the distance from the explosion center and exponentially positively correlated with the single charge. The attenuation coefficient *α* has no obvious change near 1.4 in the three directions, indicating that the geological conditions have little change in the area. The variance *r*^2^ value in the above velocity fitting curve is about 0.8, indicating that the regression value is close to the real value, and the curve fitting good.

## Study on instability and failure criterion of supporting coal pillar in end slope mining

### Study on instability criterion of supporting coal pillar

Affected by factors such as slope shape, slope angle and mining boundary, the supporting coal pillar bears the non-uniform load of the overlying strata which increase linearly with the mining depth. Due to the ‘ end effect ‘ of end-wall mining, the roof stress is concentrated in a certain engineering position before the maximum mining depth of the supporting coal pillar, rather than in the maximum mining depth position with the maximum self-weight stress. The most dangerous position of the supporting coal pillar is the position where the supporting stress of the supporting coal pillar is the largest, and the position is mainly affected by the maximum mining depth and buried depth.

Therefore, the overall strike stress of the supporting coal pillar increases first and then decreases. If the peak stress position of the supporting coal pillar is in a stable state, the other positions of the supporting coal pillar are also in a stable state. On the contrary, if the position is unstable, the supporting coal pillar is in an unstable state^14–17^. The key to determine the instability of the supporting coal pillar is the ratio of the supporting stress to the strength of the supporting coal pillar. When the ratio is less than the safety factor of the supporting coal pillar, it indicates that the supporting coal pillar has the risk of instability and failure, which is not conducive to long-term stability.

Because it is difficult to determine the actual strength of the deep supporting coal pillar in the end slope mining, it can only be inferred from the experiment combined with the relevant engineering experience. Among them, the empirical formula of CIMFR equation proposed by Sheorey based on nearly 200 cases of unstable or stable supporting coal pillars has stronger credibility:

2 $$\sigma_{zl} = 0.27\sigma_{c} h^{ - 0.36} + \left( {\frac{H}{250} + 1} \right)\left( {\frac{{W_{e} }}{h} - 1} \right)$$

where *σ*_zl_ is the ultimate strength of the supporting coal pillar (MPa), *σ*_c_ is the strength of the 25 mm cube coal sample (MPa), *h* is the height of the supporting coal pillar (m), *W*_e_ is the equivalent width of the supporting coal pillar, *W*_e_ = 2*a*, *a* is the width of the supporting coal pillar (m), *H* is the depth of the burying (m).

According to the ‘ effective area ‘ theory, the load on the supporting coal pillar can be calculated by the following equation:

3 $$P = \gamma l\frac{{a + w_{m} }}{a}$$

where *P* is the average load borne by the supporting coal pillar (MPa), *γ* is the average capacity of the overlying rock layer (kN/m^3^), *l* is the length of the supporting coal pillar (m), *w*_m_ is the width of the mining out (m).

According to the strength theory of supporting coal pillar, the safety factor *F*_s_ of supporting coal pillar is the ratio of the ultimate strength *σ*_zl_ of supporting coal pillar to the load *P* of supporting coal pillar:

4 $$F_{s} = \frac{{\sigma_{zl} }}{P} = \frac{{\sigma_{zl} a}}{{\gamma l(a + w_{m} )}}$$

The selection of safety factor of supporting coal pillar should depend on the specific stope conditions and the geological conditions of coal seam. By consulting a large number of relevant literatures and combining with the practical application experience of previous end-wall mining, the safety factor of supporting coal pillars should usually be greater than or equal to 1.3, and the safety factor of permanent supporting coal pillars should be selected from 1.5 to 2.0^18,19^. Because the physical and mechanical indexes of the related rock and soil mass in this study are relatively accurate, and the inner dump follow up immediately after the end wall mining is finished, so the safety reserve coefficient of the supporting coal pillar is selected to be 1.3 after comprehensive analysis^4,18^. Because the load of the supporting coal pillar is too complex to calculate by mathematical equation, so this paper use numerical simulation to obtain the supporting stress of the supporting coal pillar.

### Study on failure mechanism and failure criterion of supporting coal pillar

After the instability of a certain position of the supporting coal pillar, The load borne by the failure part of the supporting coal pillar is redistributed to the supporting coal pillar at both ends due to the tensile strength of the supporting coal pillar roof itself, the cohesion force of the overlying rock and the stress, the cohesion of the overlying strata and the stress. The supporting coal pillar at both ends is equivalent to the bearing sharing the gravity of the overlying strata in the failure area. The roof will not collapse immediately, and the supporting coal pillar still has the supporting ability. However, when the instability range of the supporting coal pillar continues to increase, and the load on the roof of the supporting coal pillar is greater than the tensile strength of the roof rock beam, the roof of the supporting coal pillar will collapse in a large area, and the supporting coal pillar is in a failure state. It is important to study the ultimate collapse distance of the supporting pillar and design a reasonable size parameter of the supporting pillar, which is important for the stability of the side of the open-pit mine and the improvement of the recovery rate of coal resources^20,21^.

**Fig. 4:**
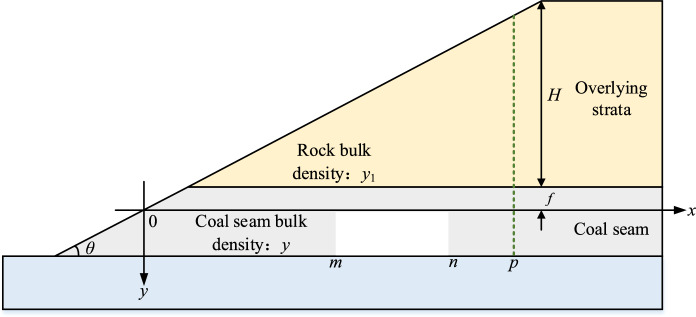
Mechanical modeling of highwall mining.

Take the junction of the slope surface and the coal seam roof as the origin, and establish the plane right-angle coordinate system, where *θ* is the slope angle (°), *γ* is the capacity of the coal seam (kN/m^3^), *γ*_1_ is the equivalent average capacity of the rock layer overlying the coal seam (kN/m^3^), *H* is maximum depth of overlying rock (m), *f* is thickness of the top plate supporting the coal pillar (m), *a* is width of supported coal pillar (m), *d* is the width of the cave (m), *μ* is the Poisson’s ratio, *m* and *n* are the start and end points of the unstable empty area supporting the coal pillar, *L*_c_ is the limit caving distance (m), *E* is the elastic modulus of coal pillar roof (MPa), *I*_z_ is the inertia moment of the rectangular section about the *z*-axis (m^4^), the mechanical analysis section of the end gang working face is shown in Fig. 4.

**Fig. 5:**
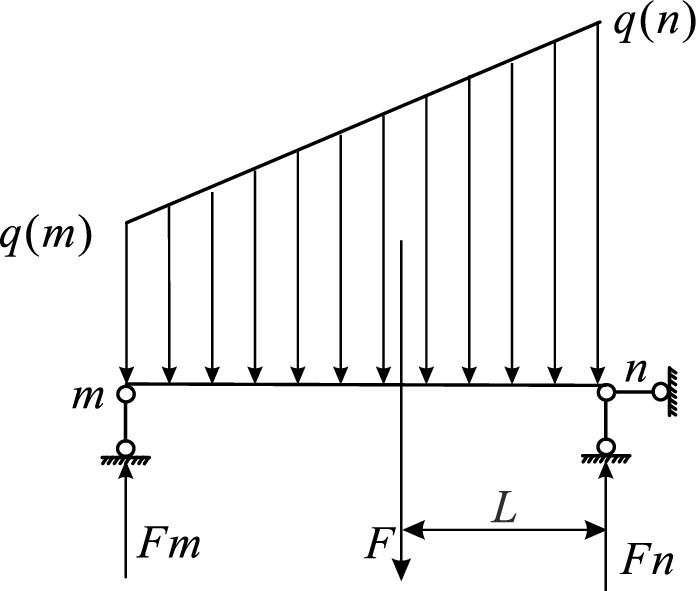
Roof mechanics model of supporting coal pillar instability area.

The overlying roof within the localized instability zone of supporting coal pillars undergoes flexural deformation under external loading, inducing compressive stress in the upper transverse micro-elements and tensile stress in the lower transverse micro-elements of the pillar cross-section. Given that the tensile strength of coal-rock mass structures is significantly lower than their compressive strength (typically 1/5–1/10 of UCS according to triaxial tests), tensile failure becomes the dominant mode of roof-pillar system collapse. According to the overall stress of the immediate roof, the roof can be regarded as a composite structure for analysis. The empty area is simplified as a single-span simply supported beam as shown in Fig. 5, and the other parts are simplified as elastic foundation beams. The analysis of the single span simply supported beam part is as follows^22^:

The relationship between *m*, *n*, *a* and *d* is^23–25^ :

5 $$m = \frac{{\left[ {a + d\left( {qm + 2f\gamma + 2\gamma_{1} } \right)} \right]}}{{3\left( {\gamma_{1} + \gamma } \right)}} + \frac{{\left( {f\gamma + \gamma_{1} } \right)\cot \left( \theta \right)}}{{2\left( {2a + d} \right)^{2} }}$$

The maximum bending moment *M*_max_ of single span simply supported beam is^26^ :

6 $$M_{max} = F_{m} - \frac{{\left( {m - x} \right)^{2} \left( {a + d} \right)\tan \theta }}{3a} + \frac{{\left( {m - x} \right)q_{m} }}{3} + \frac{2}{3}q\left( x \right)$$

After considering the effect of coal seam, the tensile strength *σ*_t_ of the roof rock beam is set. According to the strength theory of rock beam, it can be obtained^27,28^ :

7 $$\sigma_{max} = \frac{{M_{max} y_{max} }}{{I_{z} }}$$

where *σ*_max_ is the maximum tensile stress on the simply supported beam (MPa); *M*_max_ is the maximum bending moment of the single-span simply supported beam (N·m); *y*_max_ is the maximum distance of the rectangular cross-section from the neutral axis (m); *I*_z_ is the inertia moment of the rectangular cross-section about the *x*-axis (m^4^).

Because the single span simply supported beam is rectangular section, so :

8 $$\left\{ \begin{gathered} I_{z} = \frac{{af^{3} }}{12} \hfill \\ y_{\max } = \frac{f}{2} \hfill \\ \end{gathered} \right.$$

When the maximum tensile stress of the simply supported beam is equal to the tensile strength, the simply supported beam is in the critical failure state:

9 $$\sigma_{t} = \sigma_{max}$$

Combined with the above equation, the ultimate collapse distance *L*_c_ of single span simply supported beam is solved:

10 $$L_{c} = n - m$$

To sum up, When the maximum tensile stress is greater than the tensile strength of the supporting coal pillar roof, it is used as the failure criterion of the supporting coal pillar. When the length of the instability section of the supporting coal pillar is greater than the limit caving distance *L*_c_ of the roof of the mining chamber, the supporting coal pillar fails and has no supporting effect on the roof.

## Size parameter design of supporting coal pillar under blasting vibration

In this chapter, the finite difference numerical simulation software FLAC 3D is used to establish the numerical model of end-wall mining according to the geological conditions of the open-pit mine. The influence of blasting dynamic load on the stability of supporting coal pillar is simulated and calculated, and the width of supporting coal pillar under different working conditions is designed reasonably.

### Establishment of numerical simulation model under blasting vibration

**Fig. 6:**
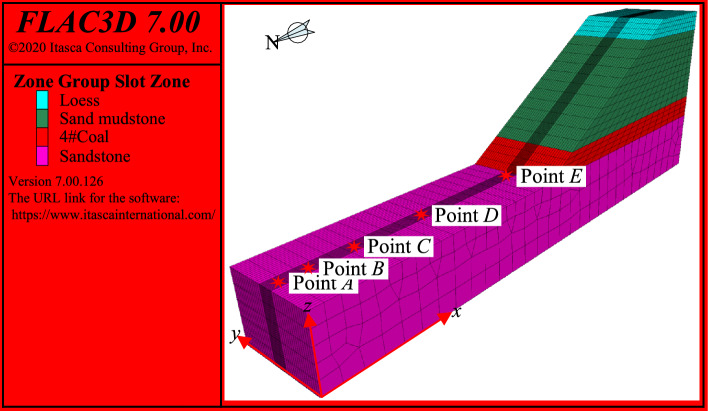
Simulation model monitoring point diagram (Version: 6.00.69, URL: https://www.itascainternational.com/).

According to the actual simulation conditions, as shown in Fig. 6, A numerical simulation model was established using the large-scale finite difference numerical simulation software FLAC3D (Version: 6.00.69, URL: https://www.itascainternational.com/). In the numerical simulation model of end slope mining, the corresponding position of *A* monitoring point is proposed, and the blasting seismic wave data obtained by vibration measurement is input. In the numerical simulation model, the corresponding position of *B*, *C*, *D* and *E* monitoring points in Fig. 1 is arranged. The seismic wave monitoring points are arranged to collect the simulated seismic wave data, and the field vibration measurement data are compared to complete the verification of blasting seismic wave and ensure the reliability of the subsequent numerical simulation results.

**Fig. 7:**
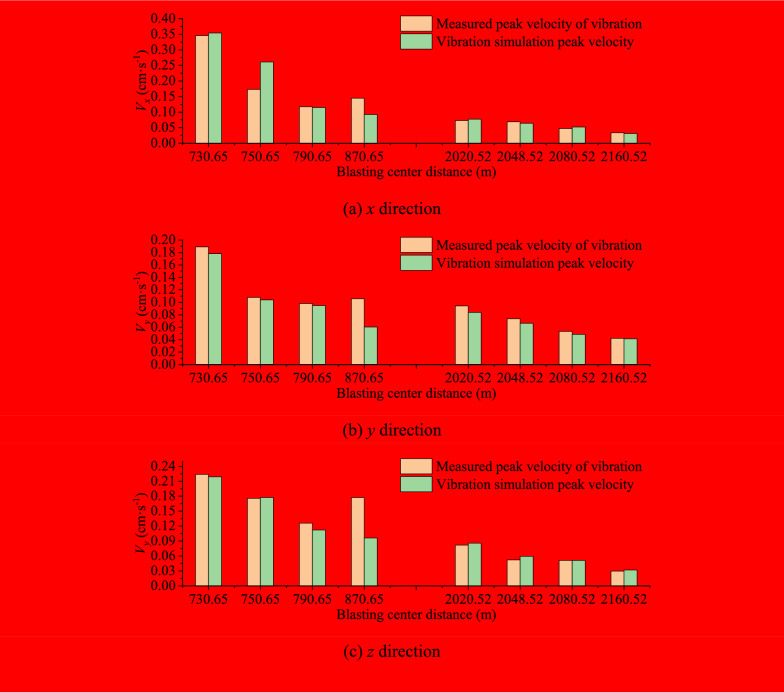
Measured experiments and blasting simulation results.

As shown in Fig. 7, a total of two sets of data are set for comparison and verification. The total error range of the two numerical calculation results and the measured results is within 10% except for some data, indicating that the attenuation law is basically consistent with the measured data, and the dynamic load simulation accuracy of the model meets the research needs.

### Parameter design of supporting coal pillar for end-slope mining under blasting vibration

#### Parameter design of supporting coal pillar under static load

**Fig. 8:**
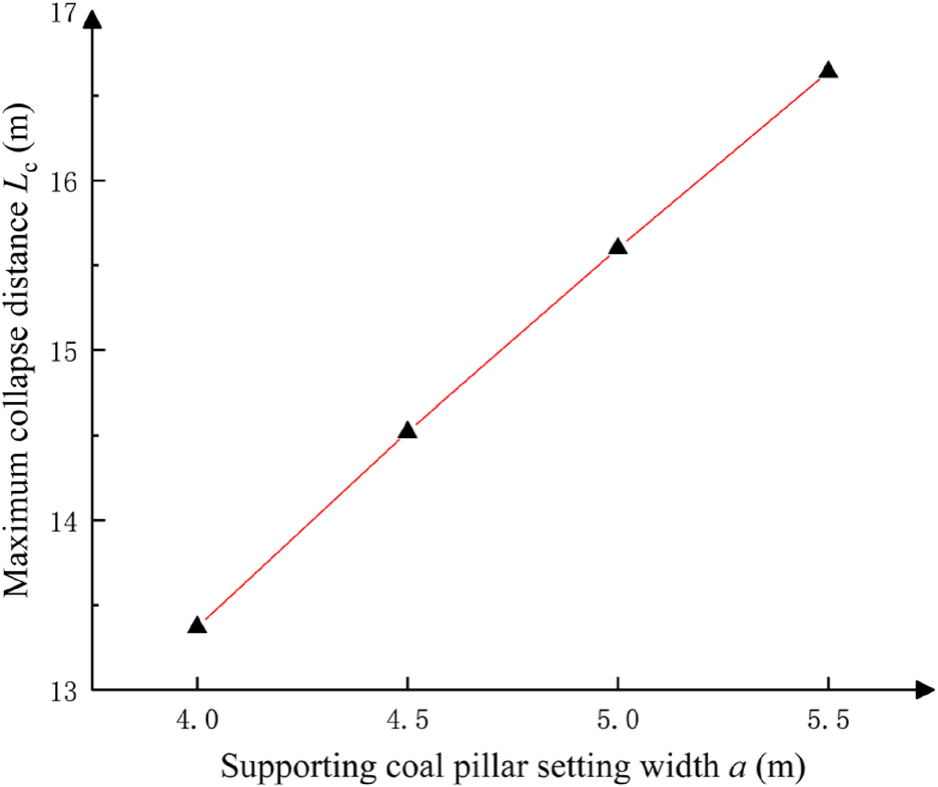
Effect of pillar width on the failure limit collapse distance of pillar.

**Fig. 9:**
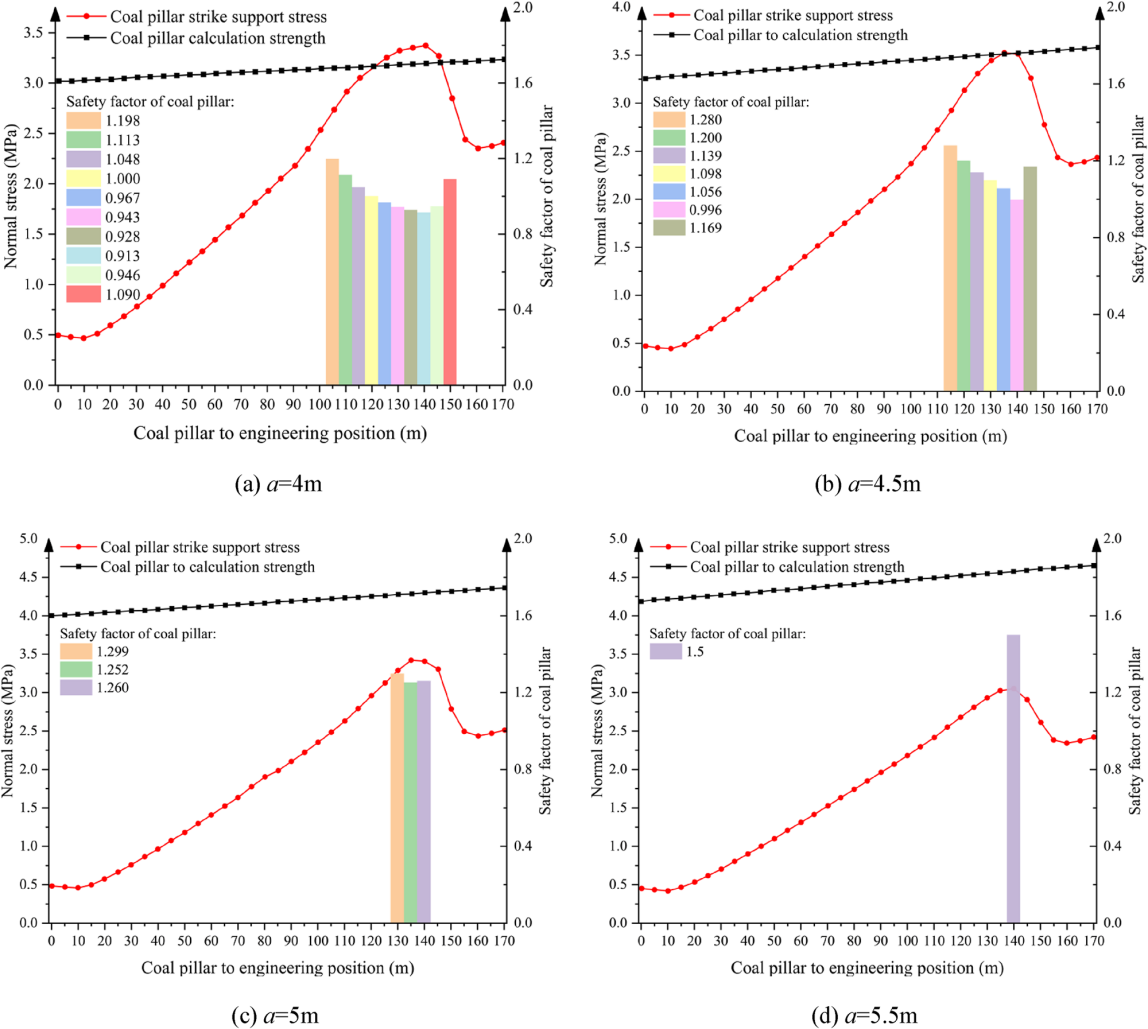
Distribution pattern of bearing stress of web pillars with different widths.

The parametric design of supporting coal pillar width was conducted based on the operational specifications of the EML340 highwall miner, which features a maximum horizontal mining depth of 150 m, cutting height of 5 m, and cutting width of 3.3 m. According to the failure criterion of supporting coal pillar, the physical and mechanical parameters of each rock layer in Table 1 are substituted into Eq. (10) to obtain the limit caving distance of corresponding supporting coal pillar under different supporting coal pillar widths, which is shown in Table 3. There is an approximate linear positive correlation between the width of supporting coal pillar and the limit caving distance of supporting coal pillar failure (Fig. 8), and the growth rate of limit caving distance of supporting coal pillar failure decreases gradually with the increase of supporting coal pillar width. Based on this, a numerical simulation model with support coal pillar widths of 4 m, 4.5 m, 5 m and 5.5 m was established to analyze the stability of support coal pillar and the variation law of support stress (Fig. 9).

**Table 3:** The limit caving distance of supporting coal pillar varies with the width of supporting coal pillar.

Setting width of supporting coal pillar *a*/m	Ultimate caving distance of supporting coal pillar *L*_*c*_/m
4	13.37
4.5	14.52
5	15.6
5.5	16.64

As shown in Fig. 9, when the mining depth is 150 m, the maximum value of the supporting stress of the supporting coal pillar along the strike direction of the supporting coal pillar should be at the mining depth of 135 m ~ 140 m. If the ratio of the ultimate strength of the supporting coal pillar *σ*_zl_ to the load *P* of the supporting coal pillar satisfies the safety reserve coefficient of the supporting coal pillar 1.3, the supporting coal pillar is in a stable state. When the instability length of the supporting coal pillar is greater than the ultimate caving distance, the supporting coal pillar is considered to be invalid. Therefore, the width of the supporting coal pillar should be set at least 5 m. At this time, the instability range of the supporting coal pillar is 15 m, which is less than the ultimate caving distance of the supporting coal pillar of 15.6 m, and the supporting coal pillar is in the state of no failure.

### Parameter design of supporting coal pillar size under different single shot charge

Based on the actual working conditions of the site, this paper sets the blasting center distance *R* = 400 m, the height of the mining chamber *h* = 5 m, the width of the supporting coal pillar *a* = 5 m, and the depth of the mining chamber *l* = 150 m. Under the parameters, four representative single-shot charges of 0.47 t, 1.24 t, 2.48 t, and 4.5 t were selected for research, and the influence of different single-shot charges on the stability of the supporting coal pillar and the change of the supporting stress were analyzed.

**Fig. 10:**
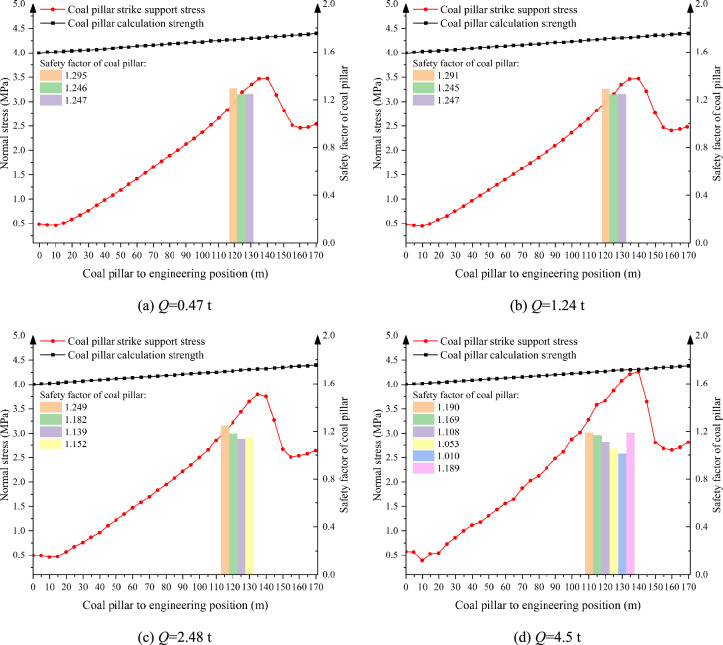
The distribution pattren of bearing stress in supporting coal pillar under the action of different single charge quantity.

**Fig. 11:**
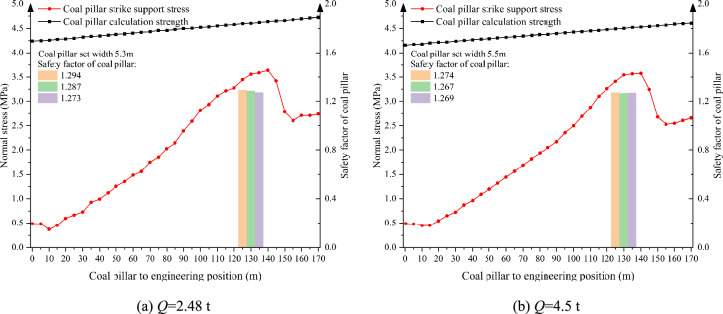
Design of supporting coal pillar width under different single shot charge.

As shown in Fig. 10, when the single charge is 2.48 t and 4.5 t, the instability range of the supporting coal pillar is 20 m and 30 m respectively, which is greater than the limit caving distance of the failure of the supporting coal pillar, and the supporting coal pillar is in a failure state. The width of the supporting coal pillar should be redesigned. The design results are shown in Fig. 11. According to the failure criterion of the supporting coal pillar, it is determined that the width of the supporting coal pillar should be at least 5.3 m when the single shot charge is 2.48 t under this working condition. When the single shot charge is 4.5 t, the width of the supporting coal pillar should be at least 5.5 m.

### Parameter design of supporting coal pillar size under different blasting center distance

**Fig. 12:**
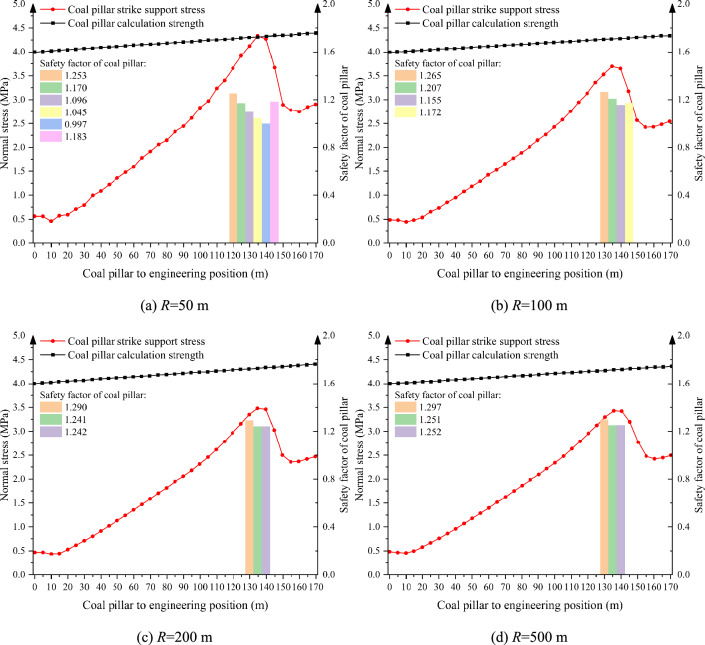
Distribution pattren of abutment stress in supporting coal pillar under different blasting center distances.

According to the Sadovsky formula, the single charge and the distance between the explosion center are the most important and direct factors affecting the blasting vibration, but the degree of influence is not yet clear. Considering that the blasting position of the open-pit mine is basically located in the north side of the open-pit mine, which is far away from the south side, there will be no close distance between the blasting source position and the supporting coal pillar position in the actual production process. Therefore, under the conditions of center-of-explosion distance, *Q* = 0.47 t, mining height *h* = 5 m, support pillar width *a* = 5 m, and mining depth *l* = 150 m, we simulated and analyzed the change of stress and stability of the support pillar when the center-of-explosion distance was 50 m, 100 m, 200 m, and 500 m (Fig. 12).

**Fig. 13:**
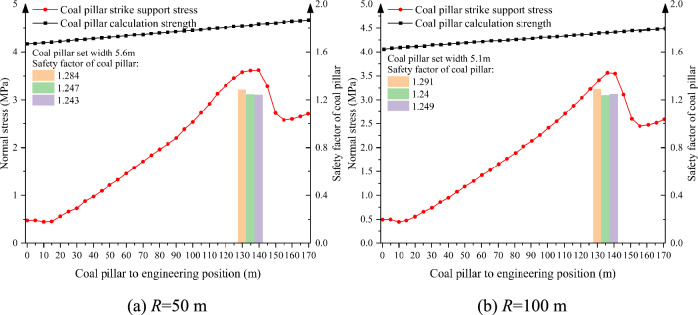
Design of supporting coal pillar width under different blasting distance.

As shown in Fig. 12, under this condition, when the bursting center distance is 50 m and 100 m, the instability range of the supporting coal pillar is 30 m and 20 m respectively, which is larger than the limit collapse distance of the supporting coal pillar failure, and the supporting coal pillar is in the failure state. So the width of supporting pillar should be redesigned, the design results are shown in Fig. 13, based on the failure criteria of the support coal pillar, to determine that the bursting center distance of 50 m, the support of the coal pillar to leave the width of at least 5.6 m; a single shot of 4.5 t, the support of the coal pillar to leave a width of at least 5.1 m.

**Fig. 14:**
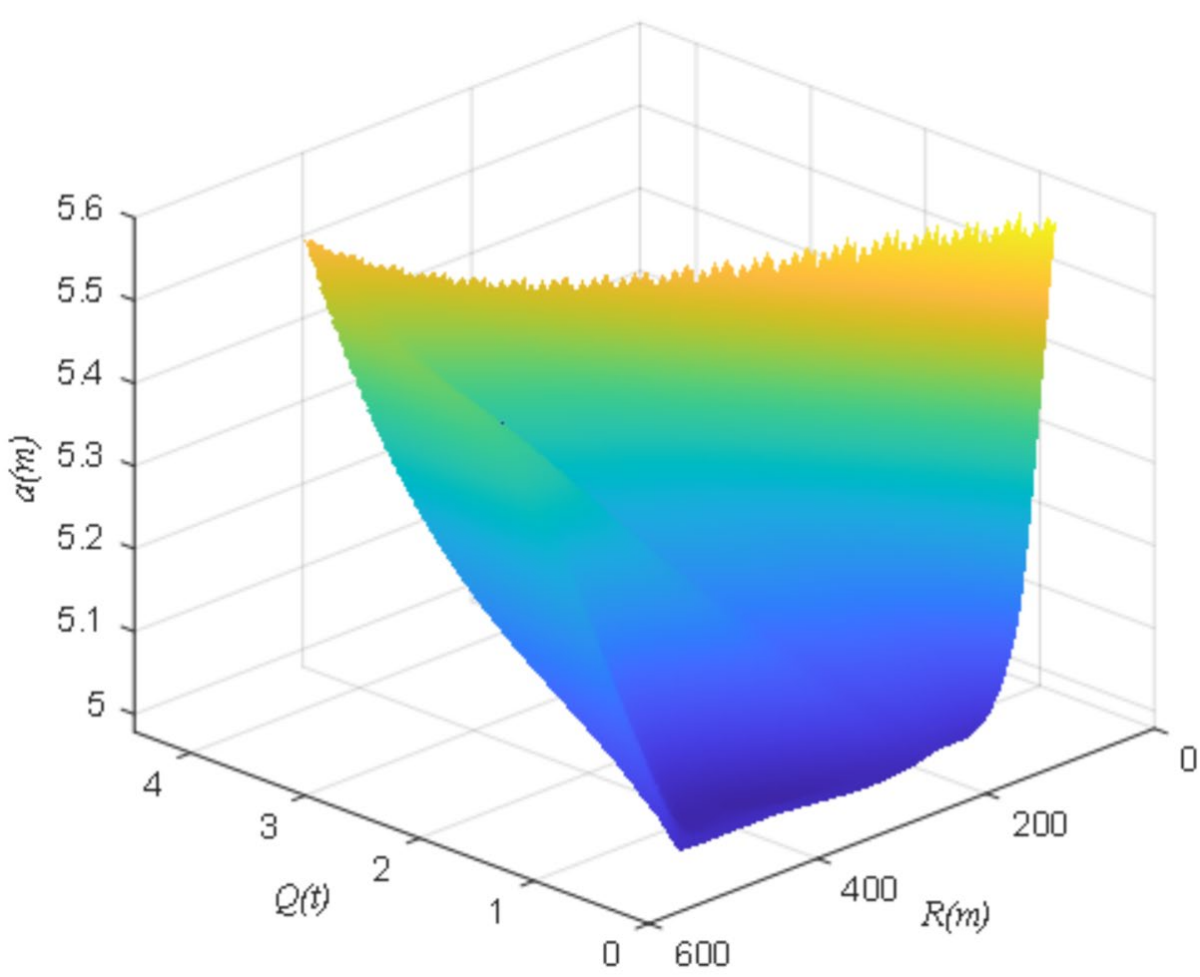
Variation law of design retaining width of supporting coal pillar under different blasting parameters.

Summarized in this paper analyzes the open pit mine in the mining cave height *h* = 5 m, the width of the support coal pillar *a* = 5 m, mining cave depth *l* = 150 m under the conditions, the width of the support coal pillar to stay with the amount of single-ring drugs and bursting center of the rule of change as shown in Fig. 14, was a nonlinear irregular surface correlation, the support of the coal pillar to stay in the width of a reasonable single-ring drug is a positive correlation with the amount of single-ring drugs, and the bursting center of the distance is negatively correlated with the relationship.

## Conclusion

This study investigates the attenuation characteristics of blasting seismic waves in open-pit mine end-slope mining through integrated field experiments, numerical simulations, and theoretical analyses. It has determined the rational width of supporting coal pillars under dynamic-static coupled loading conditions and summarized the variation patterns of pillar width with single-delay explosive charge (Q) and blasting center distance (R). These findings provide a theoretical foundation for the safe recovery of residual coal in open-pit mines and can be extended to optimize roadway support parameters and evaluate rock mass stability under dynamic loading environments. The specific conclusions are as follows:

(1) Blast monitoring data were fitted by means of non-linear regression. The peak blast vibration velocity exhibits a non-linearly decreasing decay pattern with increasing centre-of-explosion distance in the *x*, *y* and *z* directions. The main frequencies of blasting vibration waves are concentrated between 5 Hz ~ 15 Hz. The frequency of the blast vibration wave continues to decay as the centre-of-explosion distance increases.

(2) The dimensional parameters of the propped coal pillar were designed by numerical simulation of the vertical stresses along the direction of the propped coal pillar in conjunction with the instability and failure criteria of the propped coal pillar. When the roadway is 5 m in height, 3.3 m in width and 150 m in depth, the coal pillar is in a failed state if the width of the coal pillar is less than 5 m under static load. Therefore, the width of the coal pillar should be set to be more than 5 m when under the influence of blasting dynamic loads.

(3) The width of the coal pillar is approximately positively correlated with the ultimate collapse distance of the coal pillar. The rate of increase of the ultimate collapse distance of the coal pillar decreases gradually, when the width of the pillar increases. The reasonable setting width of the coal pillar is non-linear irregular surface correlation with the bursting centre distance, positive correlation with the amount of single shot charge, and negative correlation with the bursting centre distance.

## Data Availability

The datasets generated and analysed during the current study are not publicly available due the data of original blasting vibration measurement is related to mine safety and needs to be kept confidential but are available from the corresponding author on reasonable request.
